# Selections and Abstracts

**Published:** 1915-10

**Authors:** 


### SELECTIONS AND ABSTRACTS

### THE TRAUMATIC NEUROSIS.*

By Dr. Tom A. Williams, M. B., C. M., (Edinburgh), Washington, D. C. Corresponding Member Societies of Neurology and Psychology, Paris, etc., Neurologist to Epiphany Dispensary, Washington, D. C.

### PSYCHOLOGICAL FUNDAMENTALS.

Traumatic Neurosis is a complete misnomer; the condition is psychogenetic, therefore it is not neural; and the trauma is not physical. An injury in itself cannot cause a "neurosis," meaning a psychosis. This condition occurs only when the patient broods over injury, and imagines that he is a very sick person. To represent to oneself feelingly a disease, is to make oneself feel very sick, even although the disease one conceives may not itself be manifested with verisimilitude. The patient, then, having the idea that he is sick, acts so and feels so; so that after a while he actually is sick.

This is on account of the fact that the very idea of pain is capable of arousing the concomitants of pain, viz, depression of vegetative functions. This occurs because of the emotional reactions inseparable from the concepts which experience has associated with them. The situation is merely that of the dog in which Pavlow, during his experiments, suppressed the flow of gastric juice by merely showing a whip. It is a "conditioning" of a reflex, and is feasible with any dog.

This fact makes manifest how erroneous is the common opinion that the "conditioning" of affective reactions in a morbid fashion require previous morbidity for its accomplishment. This is usually stated in the formula that traumatic neurosis occurs only in the predisposed. The real factor in its induction is the momentum of the conditioning stimulus. A homely illustration is that used by the penetrating dramatist, Augustus Thomas, in "The Harvest Moon," where he makes a hard-headed lawyer, against his will, the victim of the suggestion that he is dangerously ill.

Of course, it should be obvious that the patient, although a victim of imagination, may become really ill physically; just as it is obvious that Pavlow's dog, a mere victim of imagination, is ill therewith to the extent of an incapacity to secrete gastric juice, which means very ill indeed. Indeed psychogenetic physical illness of this kind, may reach such a dergee as to cause death, as has been experimentally shown by Crile and others.

Furthermore, if the reactions have gone too far, the removal of the cause will not save the life of the animal. Short of death, secondary organic changes may occur, so that recovery will be incomplete.

But even when the stimuli are insufficient to produce organic changes the cure of the subject demands more than a mere material removal of them; for the stimuli live in memory, where they have become associated with many elements of the environment; so that the cause is not really removed until a complete reconditioning is effected of all the associational reactions which have gathered around the initial dread-bringing circumstance.

For instance, a tachycardia produced by fear, if long continued, should not be less injurious to the heart and blood-vessels than is excessive athletics; an outpouring of an excess of substances from the adrenals should just as likely produce vessel sclerosis, or exhaust the gland when it is the result of chronic anxiety as when it is due to a physiological stimulus of more direct kind; a dyspepsia or chronic constipation is just as likely to lead to malnutrition and toxemia when it is the result of mental depression as when it is due to sluggish habits or some disease.

The mechanism by which the modification of reaction occurs is usually that of suggestion. The dog which secretes gastric juice when it hears a bell does so because of the suggestion that meat will be presented him forthwith. It does not know why a bell brings meat; it mistakes it for a reaction of cause and effect, like that of a wetting when it enters the water. The process is not one of genuine reasoning. The person who is hypochondriacel after an accident has as little reason in calling accident cause and neurosis effect as has the dog in believing bell cause and meat effect. It is belief without proper ratiocination, received blindly, credulously, from someone else without criticism, that is, by suggestion. This is, unfortunately, the commonest method by which opinions are acquired by human beings. Indeed, the vast majority acquire their beliefs in no other way; and only a few scientists and still fewer superior minds have eliminated this manner of appraising the facts they encounter. It is small wonder then that the conditioning of reactions becomes morbid so easily; for we find morbidity all around. It is often dramatically impressive and frequently comes home to us by association with the deepest affections. Hysteria, then, which is merely the "effects of suggestions when these cause disease," is necessarily very widespread, and the circumstances that give rise to it and the forms that it takes, are proportional to their impressiveness, which means suggestive power, and in accordance with fashion and the zeitgeist of the country and time.

The mechanism is always the same and the victim is not aware of the systems of ideas and associated affects which constitute his psychosis.

### THE INDUCTION OF SUGGESTION PSYCHOSES. THE EMOTIONAL CONSEQUENCES. CASES AND DISCUSSIONS.

In the gross these are most clearly manifest in what our attitude of detachment easily enables us to label the superstitions of alien peoples. Thus the sufferings induced by the "gnawing fox" of the Japanese are made possible only by a deeply rooted belief in its existence. For example, a woman after labour declared she felt the "fox coming;" this was her interpretation of the after-pains she felt. The great parade by the neighbours in attempting to prevent the fox's attack only reinforced the patient's apprehension and soon a horrible convulsion signalized her seizure by the fox. Terror and convulsions held her until the exorciser was called. He declared that the fox would leave her at four o'clock the next day provided certain offerings were placed on a certain tomb for it to eat. This simple suggestion caused her to dismiss her terror suddenly at the hour designated. The crudeness of the mechanism in the case of this ignorant peasant need not make us smile, for our Western case is very little better, as the following illustration shows

It is the familiar case of an incapacitated railroad employee to whom we were called to determine whether or not there was organic disease of the nervous system. The fact that there was not is shown elsewhere in the full report of the case. The psychogenesis of the man's condition was evident in his fixed idea, due to the common belief of railroad employees that serious nervous diseases may slowly ensue upon an accident. This common belief was strengthened by the injudicious sympathy and inquiries of his friends and the doubtful prognosis of some medical men he had consulted. He "answered a thousand questions a day," he "did not know what to think about his health," and worried about his condition and circumstances; he was "too much preoccupied with his health even to miss his wife"; he had lost weight and appetite, had a sore throat, and wept much, and finally his attitude was strengthened by the lawyers who sought redress for him. He was cured within a month as a result of one interview, during which he was instructed in the role of ideas over bodily activity and the effects of worry and anxiety upon nutrition. In the certificate it was stated "there is and has been no disease of the spinal cord or peripheral nerves at play in the induction of any of the symptoms which I find. The erroneous belief that there has been such an injury powerfully contributes to the anxiety which maintains his present state."

The role of the idea of "shock" in perturbing this man's emotional life is strictly comparable with that of the gnawing fox of the Japanese folklore. In both cases too there was the period of reflection and incubation of the morbid notion, a familiar feature of such cases which has been insisted upon by Souques. It is rare that the symptoms ensue until after a time of meditation, during which the complex is systematized.

The cure was not so simple as that of the Japanese exorcist. But it was a definite one, for the railroad brakeman was taught to understand the mechanism of his affection and thus to overcome any future harm from the credulity in which he had grown up.* The Japanese woman, on the other hand, remained liable to another attack, as her belief in the fox was only reinforced by the manner of its removal.

A contrasting case where therapeutics failed will further push the lesson home. A government employee was injured by a falling case and remained barely able to walk even after his bruises of head and shoulder had healed. Called in consultation we explained the mechanism of his present incapacity and directed how to remove it. His family physician's acquiescence to our directions was only formal, as his bent was not psychological enough to grasp the principles at work. The fearful solicitude of the man's wife, too constantly reinforced his timidity, so that in spite of a considerable temporary improvement he did not progress to full recovery, but remained lachrymose, depressed and relatively incapable on account of the persistence of his false belief about his health and powers.

Loss of "Nerve" After an Accident. Again, a railroad freight conductor was sent to me for care from North Carolina by the Southern Railway Company. He had fallen off a truck and had been much shaken and bruised. Unlike most persons in such case he did not complain of pain or paralysis, but merely stated what was truth, that he "could not sleep, and remained in a state of nervous agitation which would even cause him to cry at times and made life unbearable, from incapacity, weakness and mental depression, so that he felt utterly unable to return to work, feeling that he could not perform it." In his happy domesticity, there were no extrinsic psychological factors except the mental habitus of hyperconsciousness of ambitious type. There was an entire absence of roughness often seen in men of that occupation. In its essence, the situation was that the man felt unable to, and did not want to, lead again the arduous life of a railroad man, for which he was in reality temperamentally unsuited.

But even in this case adaptation to the unpleasant environment might have been accomplished had not the pernicious influence of the struggle for indemnification preponderated so as to interfere with psychological reconstruction. Instances of this kind are numerous. Isolated examples spring to the mind of every railroad surgeon; but an extensive comparison between cases equal in value to a deliberate experiment is best afforded by the observation of a train wreck in which 200 passengers were injured, about half of them severely. Only about 20 of these passengers developed traumatic neurosis. Some of these received heavy damages, upon which their health was immediately restored. In one case, however, a cure was effected by Dr. Bevan, the observer, without recourse to a lawsuit; but even this was done against the active protestations of the patient and only by extraordinary perseverence and determination on the part of the physician, whose method of persuasion was so insistent as to make the patient weep.

COMPENSATION ACT CURATIVE.

But it is not always that the indemnity effects the cure. There is a case well known in Washington where $17,000 was allowed by the court to a man in whom a street car accident induced the belief that he was incapable of locomotion. This lasted for seven years, during which the patient went about in a wheel-chair administered to by a solicitous wife. His wife's belief that he could not walk was rudely disturbed after the plaster had fallen from the roof while they were asleep in bed one night, when she found her husband seated in the corner of the room twelve feet from the head of the bed. She argued that if he could walk while asleep at night he could walk while awake by day. This he did in trembling fear after insistent persuasion by her, and eventually recovered in a few days.

It would be idle to pretend that these were deliberate simulators for gain. They were honest pretenders, just so much as is any genuine hysterical the victim of a suggestion that he is incapacitated. These cases must be carefully differentiated from those who intentionally imitate symptoms in the hope of gain, even although the gain be merely the sympathy, attention, or notoriety from other people. The first type we call malingerers, the second mythomaniacs, fantasticals, or pathological liars; neither of these types has immunity from the psychological reaction of suggestibility, which may eventuate in the genuine medical hysteria.

The initiator of symptoms may, of course, be an actual accident or disease; and equally so it may be derived from the mere idea of injury or disease, so that the clinical problem to be analyzed may be exceedingly complex. Besides this, the patient may refuse access to his psychological groundwork, perhaps merely on account of shyness or shame of thoughts concerning which he fears misunderstanding or ridicule, end entirely apart from any question of venality. This phenomenon is found very commonly in psycho-analytic work and it is the shame-faced reticences of patients which have led to the need of the use by some analysts of mechanistic expedients, such as the association-experiment (Jung,) the dream unravelling, and the free-flow association.

These genuine psychoneurotics are entirely curable, quite apart from any question of indemnity in itself; but the struggle for indemnity cannot be given up without loss of self-respect in the implied confession of dishonesty or at least of the gross error of psychological interpretation concerning the role of the accident itself as the provoker of the illness; so that there is a preoccupying search by the patient for facts to ratify his belief that the accident had damaged him. This inevitably loads to imaginations, exaggerations, and falsifications inevitable for a mind not scientifically trained. It is only when the patient has a glimmering of his mistake that he begins in desperation to defend it by conscious self-deception in order to bolster up a psychosocial attitude, the negation of which would, he believes, be derogatory.

The construction of this state of mind is thus described in my article before the 1913 International Congress of Medicine.

"Especially prone to this damaging sequence are persons whose imagination has been made rampant by the cultivation of the credulous fears of childhood; their fear-reaction to that which they do not understand is a dominant one, and they are easily beset by an idea linked with fear. The commonest of the fears which result from accident or injury is that of bodily harm. It is difficult for a person of this type, when ignorant of his own structure and functions, to shake off the foreboding created by an impressive catastrophe; and it must not be forgotten that what others regard as trifling the victim may look upon as catastrophe, judged by its possible effect on him. Prepossession by the idea of one's own disability is an inevitable consequence. This leads to abstraction from and inattention to the affairs of ordinary life which, if not trifling by comparison in the patient's mind at least cannot claim the attention properly needed. Hence ensues the well-known diminution of the capacity to think, work or take part in social life. This incapacity, when the patient becomes aware of it, leads him to still further accentuate the result of his injury and thus to augment his alarm about his health. Thus is constituted the vicious circle of hypochondria. Even a nosophobia may ensue, such as the fear of lost manhood, insanity, paralysis. Alarm at this impending disaster must, of course, be distinguished from the primary alarm due to the accident itself."

FORENSIC PRESENTABILITY.

This mechanism is so simple that it can clearly be grasped by any intelligent person even without medical training, it has been convincingly popularized by Mr. Addington Bruce in "The Outlook," of May 9, 1914.

There should be no difficulty, then, in convincing a court of the nature of these cases and of their cureability when properly dealt with; and this should lead to an equitable appraisement for damages for loss of time and distress of mind. That this is not at present the case, we believe to be due to the want of knowledge of many expert witnesses concerning the whole subject of the psychoneuroses. The point of view here set forth is entirely foreign to a mind of which the habit has been to be content with the farrage of confusions which was all that the older text-books afforded concerning functional nervous affections. Besides this the appraisement of the functional nervous affection is inextricably interwoven with the whole subject of neurological diagnosis. A physician who is not thoroughly conversant with modern neurology is sure to give the jury a false impression of the status of his case unless he strictly confines himself to concrete facts observed by himself and leaves their interpretation to the neurological expert who follows him. In the conduct of such cases, counsel should entirely discard the antiquated and misleading accounts in text-books of medicine and neurology; the issue should be presented solely by concrete facts elicited from the patient himself as observed by medical men and others, and the interpretation of these aside from all technical concepts, in the plane of simple good sense, which means good psychology. A striking instance of this error has just transpired in the District of Columbia, where a case carefully outlined on this basis was thrown into confusion by the introduction of a general practitioner who, without adequate knowledge, took it upon himself to instruct the jury concerning neurological and psychopathological interpretations of which his hazy knowledge was only second-hand.

It was the case of a woman who after a trifling glass cut and a knock on the elbow gradually developed a complete flaccid paralysis of the right arm. The allegation of neuritis was easily disproved; the joints were not diseased; the spinal cord was intact, and the brain centre was not damaged. These were shown by complete mobility, absence of wasting, presence of the reflexes without exaggeration or spasticity. The psychogenetic nature of the paralysis was very clear, and the patient's own physician fully admitted this in the witness box, and also that such paralysis often occurred without trauma of the body at all. Facts of this kind are really not difficult to demonstrate to a jury if a properly trained psychopathologist is employed.

This was demonstrated very clearly in the case of Hill vs. Chicago, Milwaukee & St. Paul Railroad, at Redwing, Minnesota, December, 1911. Here the psychogenetic nature of an affection which simulated lameness alleged to be due to sacro-iliac and lumbar injury, was so clearly shown the jury, that instead of $15,000 damages demanded the allowance given was merely $1,500 for loss of time and stress of mind. This was a particularly "dangerous" case from the forensic aspect; but the railroad had become weary of paying indemnities for psychological accidents, and made this a test case. The crucial point depended upon a demonstration by the neurologist that the attitude and movements of the patient did not conform to a syndrome which should have been present had the injury been where believed. This, along with the integrity of the reflexes and lack of wasting, convinced the jury that no lesion was present.

In conclusion, let me emphasize the importance of the fact that functional nervous syndromes which occur after accidents differ in no way from those found in persons who have been subjected to no accident at all. The accident then was a red herring across the trail of the real cause of the psychoneurosis. These doctrines have already been elaborated by me with numerous illustrations.

1705 N. Street.

Traumatic Neurosis and Babinski's Conception of Hysteria, Transactions of Congress of Industrial Actions. Rome, and Medical Record, 1909.

Successful Psychotherapy of Traumatic Neurosis, Amer. Jour. Surg., 1909.

Idea and Affect in Traumatic Neurosis, Jour. Abnor. Psychol., 1910; also Charlotte Med. Jour.

Psychic Effects of Accidents, Transactions of Southern Railway Surgeons, 1912, and Monthly Cyclopedia.

Occupation Neuroses, International Congress on Hygiene, 1912; Medical Record, 1913, Transactions of International Congress of Medicine, 1913; Cleveland Med. Jour., 1914.

Treatment of Hysteria, Jour. Amer. Med. Assoc., November, 1912.

Hysteria and Pseudohysteria, Amer. Jour. Med. Sci., September, 1910.

Mental Healing, British Med. Jour., Vol. II., 1913.

Faith Cures and Genuine Psychotherapy, Illinois Med. Jour., Oct., 1914.

---

### A SCIENTIFIC SUBSTITUTE FOR THE DEATH PENALTY.

JOHN S. DURAND, ESQ., NEW YORK.

It is not intended within the limits of this paper to discuss the right of organized society to take human life. Organized society does take human life and it is generally considered as having that right, either for its own protection or by way of punishment for certain crimes.

"Whoso sheddeth man's blood, by man shall his blood be shed: for in the image of God made he man," was written four thousand years ago at a time when the waters which covered the earth had assuaged, and at divine command Noah had gone forth from the ark and every living thing with him to replenish the earth, and since this divine sanction of the law of life was promulgated, the rule of a life for a life has been observed to a greater or less extent throughout the world.

In early times the individual exercised the right to require for the life of his kinsman the life of his neighbor who slew that kinsman, and as men gathered together in families and tribes the family or tribe avenged the death of one of its members killed by a neighboring family or tribe. Later on money or property was frequently accepted in lieu of the life demanded, and finally organized society as a collective body asserted the exclusive right to execute retribution upon neighboring nations by war and punish its own citizens for crimes committed by them, or by others within its territorial jurisdiction, on the theory that the crime was an injury to the sovereign power which represented the entire community.

Thus prosecutions for offenses forbidden by federal statutes are brought in the name of the United States and proceedings in the State of New York to punish a criminal act under its laws are instituted by and in the name of the people of the State of New York. In Pennsylvania, the constitution provides that all "prosecutions shall be carried on in the name and by the authority of the commonwealth of Pennsylvania, and conclude against the peace and dignity of the same."

So also organized society asserts the exclusive right as a if committed within its jurisdiction and to prescribe what penalty shall be inflicted for the punishment of these crimes.

The theory of modern criminal statutes is that they shall be corrective and their sanction is intended to act as a deterrent rather than as a punishment. The idea theoretically is that the consequences which the law says shall follow any prohibited act shall be calculated to prevent the commission of the act, and in the case of minor offenses if the act be committed, shall tend to reform the evil doer and show him the iniquity of his ways. Wilful murder, however, has always been in a class by itself and regarded as such a serious offense that the state or nation to define the acts which shall constitute crimes life of the murderer or his "legal death" by life imprisonment has been required by society in expiation of the crime, and as an example best calculated to deter others from the wilful taking of human life.

Under present conditions when society forfeits the life of a criminal, he is put to death usually by hanging or elecrocution. and much of great value has been learned from electrocutions as to the effect of electric currents of high voltage upon the human system, but there is an almost limitless field of research as to the effect of various drugs upon the human body and as to the possibility of so controlling its functions that operations now considered impossible of performance may become a matter of every-day occurrence. Great strides have been and are being made in surgery. It is reported that the heart of an animal has been stopped for two and a half minutes without any trouble following, but as yet there has been no opportunity to test the method on man.

In the medical field new drugs are being discovered and new serums prepared, the effects of which upon the human system are yet to be determined. Much might also be learned as to the effect of many drugs now in use, on the blood of human beings, and as to the various processes of digestion, assimilation and elimination. It remains to be determined whether or not the blood can be diverted from the veins, and, driven by a small electric pump, forced through a filter bed inhabited by minute species inimical to disease germs in the blood, or whether some artificial means cannot be devised for the mechanical increase of the cocites in the blood. With a properly constructed machine, various poisons could be introduced directly into the blood and their effects noted while the blood itself is passing through glass or other transparent tubes as living blood, the small portion so treated being switched out of the artificial circulatory system and not returned to the body. An appropriate moving picture camera properly adjusted would record the various changes in the composition of the blood. Such a camera would also record the changes caused by drugs administered internally. Machines now in use would register the action of the heart under the influence of various stimulants and narcotics. The effect of the x-ray, the n-ray, the ultra violet ray and all the other rays yet to be discovered, upon human tissues and human nerves could be studied, the action and reaction of human neves under stimulants and sedatives could be determined, and the field of human knowledge as to man and his body immeasurably increased, if the subject of these investigations could be obtained.

How much more scientific, therefore, instead of blotting out a life that is forfeit to the State, to utilize that life in the interest of science and for the purpose of assisting man in his fight against disease and for the relief of human suffering.

Startling, you will say—perhaps cruel. Yes, but it is not cruel to put a man on the gallows or in the electric chair and take his life to no purpose other than to deter others from the commission of the crime of which he has been convicted, and in States where there is no capital punishment it is not cruel to deprive a man of his liberty so long as he shall live, and decree him to be "civilly dead?" When we speak of punishment, we must differentiate between cruelty which is wanton and the infliction of suffering for a justifiable end. Under the plan now suggested, there need not be, nor should anything be allowed to be done calculated to inflict torture or any real suffering. Men have lived for years with a fistula, without experiencing any serious inconvenience, but these men being physically and legally alive could not be compelled to submit themselves to observation.

To compel a man to lie in practically the same position for several hours need not cause any suffering. Drugs and serums which have been perfected as far as possible in experiments on animals so that they are believed to be harmless to man, can be tried on a human being without serious pain or inconvenience. Sufficient human blood can be taken to completely replace the blood of a rat or a rabbit without danger to the man, although the consequences to the rat or the rabbit might be disastrous, but if the human blood which completely replaced the blood in the small animal retained its characteristics, which are materially different from those of the animal's blood, much invaluable information could be gained through experiments on the animal. In fact, there is a limitless field for scientific investigation without cruelty to the individual, or suffering on his part, which would result in the greatest benefit to mankind, and to incarcerate a man for life for the purpose of scientific investigation, would probably be as great a punishment to the individual himself and as great a deterrent to others as the death penalty.

Of course, the investigations to be made would have to be in the hands of men of the highest standing. Each test should stand alone, and an application for permission to make it must be made in writing describing fully what is proposed to be done and a certificate for that particular test must be issued for each particular test which should be made in a room or place to which medical and scientific men, and others who are not influenced merely by morbid curiosity, should have access.

Vivisection, as ordinarily understood, certainly would not be permitted, but a commission should, after careful investigation, decide the proper limits within which tests could be allowed, always bearing in mind that nothing calculated to inflict torture or probably calculated to cause death should be permitted. This would adequately protect the prisoner from suffering and prevent public critiicsm on humanitarian grounds.

Undoubtedly some would object to the plan proposed on the ground that it would be cruel and inhuman. Many of those persons now object to all tests and experiments on animals. It is a fact, however, of which there is abundant proof, that tests on the lower animals have resulted in the greatest good to humanity. Smallpox has been practically stamped out. Diphtheria has lost its terrors. Typhoid fever, which killed more soldiers in the Spanish war than bullets, is now under control. Yellow fever has been conquered at the expense also of human life voluntarily offered as a willing sacrifice, in the search for the cause and the cure of this dread disease. These tremendous achievements have been accomplished as the result of experiments, which at the time they were made might have been and probably were characterized by some people as cruel, cruel, inhuman and barbarous. But cruelty does not consist in the infliction of pain, but in the "disposition to give unnecessary pain to others," and the question whether or not pain is necessary depends upon the object in view and the merit of that object, and that in turn depends upon the state of mind of the community. It cannot be laid down that the infliction of any pain is cruelty. To drive a hypodermic needle into the arm or body of a child, without reason, would be cruelty, but if the pain be inflicted to save the life of the child by checking the ravages of diphtheria, or anticipating an indicated attack of that disease, the act would be the most humans possible, but if the amputation be indicated as the only means of saving life, to refrain would not only be a cruelty but also would subject a medical man to charges of incompetence or malpractice. There is no operation of ancient or modern times, and no drug or other substance has ever been discovered for the alleviation of human suffering, which has not been tested for a first time on some human being, possibly without his knowledge or consent, and without the knowledge or consent of his relatives or friends, and the grave hides many tragedies due to the lack of exact knowledge on the part of those who sought groping in the dark for the light that would bring health to the sick and the maimed. But there are other graves and they hide tragedies decreed by the state. Had another course been taken, there would have been but one grave, a milestone in human progress, or perchance no grave at all, until in course of nature the condemned man having by his aid in the humane search for the yet unknown, fully expiated his crime against his fellows, lays himself down to his last long sleep in the consciousness that after all he has not lived in vain, and that the world is on the whole better off because he has lived. How much better than an ignominious death upon the gallows! How much greater the conservation of human life!

But if the infliction of necessary pain upon an individual for his own good is justifiable, why should not at least necessary amount of pain be inflicted vicariously for a justifiable end? Nations send the flower of their manhood to war with the absolute knowledge that many will be killed, many more frightfully mutilated and maimed, and many, many more be claimed by disease, and when the grim destroyer has done his deadly work, we meet to dedicate a portion of a great battle field as the final resting place of those who died that the nation might live, and each one of those grassy mounds and every one of the white tablets which strew the hillside tells its story of an innocent life given for the general good. The battles of peace are far more important to humanity than armed conflicts, and why should not those who have sinned so grievously against their fellow men that society has hast them out, be conscripted and put on the firing line?

It remains only to consider whether or not such a plan would infringe the constitutional rights of the prisoner under the Federal and State constitutions. If a law were enacted either condemning the convicted prisoner to imprisonment for test purposes or to death with the option of such imprisonment, and the trial be had according to the course and practice existing in all similar cases, he could not be heard to complain that he had not had the benefit of "due process of law." That clause of the Constitution does not relate to the punishment for crime, but to the law of the land as to the various steps to be taken and the procedure to be observed in ascertaining if the accused should be punished in the manner all others convicted of similar crimes are punished.

Even if a prisoner be subjected to various experiments, it could not be said that he was "twice put in jeopardy" for the same offense. This provision refers to the trial of the accused and not to the punishment. There have been several cases where a scaffold rope has broken. The prisoner did not because of that accident become entitled to the benefit of the constitutional provision last quoted, and as soon as a new rope could be procured he was duly and legally executed. But a third constitutional provision which forbids the infliction of cruel and unusual punishments, presents a question somewhat more difficult of solution.

This clause is borrowed from the Bill of Rights passed in the first year of the reign of William and Mary, 1869, being Chapter 2 of the Statutes of that year. It read: "Excessive bail ought not to be required nor excessive fines imposed, nor cruel and unusual punishment inflicted." When this statute was made part of our Constitutions the word "shall" was substituted for the word "ought."

While in the Bill of Rights the provision referred to was probably only a declaration of the principles which the English people wished to have observed in the conduct of the government of the foreign prince they were about to place upon the throne made vacant by revolution, it is probable that the character of the punishments in vogue when our constitution was adopted by the colonies induced the framers of the Constitution to change the word "ought" to the word "shall." The Bill of Rights did not, however, serve to mitigate the severity of the punishments in vogue when it was enacted, but those punishments continued to be inflicted long after the enactment of the Bill of Rights. These punishments are graphically described in the case of Done vs. The People, 5 Parker, page 382, where the court says:

"The punishments in England, when the offender was to suffer death were inflicted in various ways. Under the direction of military courts he was shot. When condemned by ecclesiastical tribunals; he was not unfrequently burned at the stake, as if his priestly judges designed that the heretic, on going out of this world, should have a foretaste of the punishments to which they also consigned him in the next. For treason against the state the great sword of justice was to fall. The condemned man was sentenced to be hung, taken down while still alive, beheaded, disemboweled and quartered; with few exceptions, however, the axe of the executioner only was used, and the criminal was simply beheaded. If, however, in case of high crimes, especially treason, the prisoner stood mute and refused to plead, he might be sentenced to be pressed to death, a punishment inflicted by placing the prisoner on his back, naked, in a cold dungeon, with his arms and legs extended by cords to the four corners, and with iron or stone laid on his breast, and then left till death from cold, or pressure, or exhaustion, came to his relief. Lastly, for the crime of murder and numerous other felonies, the criminal was sentenced to be hung by the neck till he was dead." These extreme punishments had gradually fallen into disuse, for the court continues: "In New York we had no conviction, so far as I am informed, of any person charged with heresy. In 1705, during the administration of Lord Cornbury, the Rev. Francis McKemie was indicted and tried in the city of New York for preaching without the Queen's (Anne' license. The trial was not in an ecclesiastical court, but in the Supreme Court. McKemie was a Scotch Presbyterian, and he defended himself, maintaining with marked ability that preaching the gospel was no crime at the common law; and the jury, notwithstanding the marked partiality of the court against him, rendered a verdict for acquittal. A few years prior to that, Colonel Nicholas Bayard and Alderman John Hutchings were tried, also in the city of New York, on indictments for high treason. Both were convicted and sentenced to be hung, emboweled and quartered. They were among the most eminent citizens in the province of New York, and their trial and conviction were, as they had often been in England, the fruit of party rancor and judicial corruption. The sentences were afterward reversed by directions sent out fro mthe English government. With the exception of the trials of Leisler and Milbourne, both of whom were convicted and executed by hanging, these, I believe, were the only trials for treason in New York while a province. The extreme punishment of emboweling and quartering was never inflicted. Of burning at the stake we have several melancholy instances—none, however, for heresy. This punishment was inflicted, so far as I have been able to ascertain, upon oppressed and despised races. Thus, in 1707, Lord Cornbury, governor in writing to the Board of Trade in London, says that 'a most barbarous murder has been committed upon the family of one Hallet by an Indian man slave and a negro woman,' and he adds, 'I immediately issued a special commission for the trial of them, which was done, and the man sentenced to be hanged and the woman burnt, and they have been executed.'

"In 1712 Robert Hunter, then being governor of New York, in writing to the Board of Trade, among other things, speaks of a slave insurrection and the killing of several whites by the negroes. He says the slaves were arrested and 'forthwith brought to their trial before the justices of this place, who are authorized by act of assembly to hold a court in such cases. In that court were twenty-seven condemned, whereof twenty-one were executed; one being a woman with child, her execution by that means suspended; some were burnt, others hanged, one broke on the wheel, and one hung alive in chains in the town; so there has been the most exemplary punishment inflicted that could possibly be thought of.' He might well have added, as he did, 'which only this act of assembly could justify.' (See Vol. 5, Colonial His., pp. 39, 341.) In 1741-42 there occurred what has become historically famous in this State as the negro plot, wherein love of freedom in the negro, introduced of popery, incendiarism and plunder are combined. Thirteen blacks were burnt at the stake, and one white man and one negro were gibbeted. (See Pamphlet containing trial, and Smith's History of New York, Vol. 2., pp. 70-72.) About 1772, or 1773, a negro man was burned at the stake at Johnstown, then the county seat of Tyron County, for a rape committed on a white woman. There may be other instances, but those mentioned are enough to require in the (American) bill of rights that neither 'cruel nor unusual punishments shall be inflicted.' "

Historically, therefore, it appears that punishments which at the present day would be regarded as inhuman and barbarous were inflicted long after the English Bill of Rights was enacted. From the preamble to that statute which recites that excessive bail has been required, excessive fines imposed and "illegal and cruel punishments inflicted," it is plain that the statute was directed against the illegal and arbitrary acts of James II and his puppet judges, and as a declaration that under the joint sovereignty of William and Mary, to whom the crown had just been offered, no punishments except those prescribed by parliament or such as were then in vogue should be permitted.

The Constitution of the United States as originally drafted and ratified by the States did not contain the provision forbidding cruel and unusual punishments, but that provision was added as one of the amendments subsequently adopted, which have been characterized as 'our national Bill of Rights, and apply to each department and to all classes of officials." The provisions of those amendments have been largely copied in the constitutions of the various States, but different States still have different punishments for the crime of murder. In some the convicted man is electrocuted, in others hanged and in others imprisoned for life, and it is fairly to be inferred that in States where life imprisonment is the extreme penalty it is regarded as cruelty to put a man to death by hanging or electricity, and that in States where hanging is decreed, and the malfactor in many cases slowly strangles to death, electrocution is not considered more humane. As in any case the malfactor is not deprived of any constittuional right, it would seem to follow that whether or not a punishment is cruel and unusual, and therefore obnoxious to this provision of the Constitution, is largely a matter of the opinion held by the citizens of the State which inflicts the punishment.

Comparatively few decisions have been rendered under this clause of the Constitution forbidding cruel and unusual punishment, although it has been considered to some extent by the text writers. Thus Cooley in the Principles of Constitutional Law, 3rd ed., p. 324, states as follows:

"What punishment is suited to a specified offense must in general be determined by the legislature, and the case must be very extraordinary in which its judgment could be brought in question. A punishment may be unlawful either (1) because it is in excess of, or different from, that prescribed by law; or (2) because it is not warranted by the judgment of any competent court; and possibly (3) because, though apparently warranted by law, it is so manifestly out of all proportion to the offense as to shock the moral sense with its barbarity, or because it is a punishment long disused for its cruelty until it has become 'unusual.' Nothing more definite can on this point be affirmed."

The decisions of the courts have usually proceeded on the theory that the Constitution forbids the barbarous punishments in vogue prior to the enactment of the Constitution, some of which have been referred to above, but that the legislature, being a representative body and supposed to reflect the sober sentiments of the people, has in its discretion the power to prescribe and change the mode of punishment for crime, but not to inflict manifestly cruel and unusual punishments, as burning at the stake, crucifixion, breaking on the wheel or the like. This, in effect, was held in People ev rel Kemmler v. Durston, 119 N. Y., at page 578, and affirmed by the United States Supreme Court in 136 U. S., page 436 (where the contention was made that electrocution was a cruel and unusual punishment), for the court says:

"If it cannot be made to appear that a law is in conflict with the Constitution by argument deduced from the language of the law itself or from matter of which a court can take judicial notice, then the act must stand. The testimony of expert or other witnesses is not admissible to show that in carrying out a law enacted by the legislature, some provision of the Constitution may possibly be violated."

The principle, of course, only applies to acts of the legislature and not to ordinances or other regulations of subordinate jurisdictions. It was so held in the case of Ho Ah Kow vs. Nunan, 18 Am. Law Reg. (N. S.) 676, where an ordinance of San Francisco provided that every male person imprisoned in the county jail should immediately have his hair cut or clipped to an uniform length of one inch from the scalp, and under this ordinance the sheriff cut off the plaintiff's queue, and the court held that as the ordinance was expressly directed towards the Chinese, and that a treatment to which disgrace is attached and which is not adopted as a means of security against the escape of the prisoner (or for any sanitary reason), but merely to aggravate the severity of his confinement, can only be regarded as a punishment additional to that fixed by the sentence. "If adopted in consequence of the sentence it is punishment in addition to that imposed by the court. If adopted without regard to the sentence, it was wanton cruelty," and the court declared the ordinance unconstitutional, as repugnant to the constitutional provision forbidding cruel and unusual punishment, but intimated that the ordinance would have been sustained if its object had been a proper one.

It is to be observed that in the case last referred to the court permitted testimony to be introduced showing that it was a disgrace and cruelty to a Chinaman to have his queue cut off under an ordinance without adequate sanitary reason or for the purpose of identification as practiced by the State in the penitentiaries, but this ordinance was not a law passed by the legislature. If it had been, the court would doubtless have been governed by the principles stated in the Kemmler case referred to above, provided the reason for the statute was apparently justifiable.

On the whole, it seems to conclude that any law changing the death penalty to imprisonment for life for observation purposes which the legislature of any State representing the public sentiment of that State might pass, would justify itself and be held to be constitutional. There would therefore seem to be no reason on humanitarian or other grounds why the criminal, who by reason of his own acts has forfeited his life to the State, should not be used humanely and without torture for scientific study and investigation for the purpose of prolonging the lives and reducing the sufferings of all mankind, and hastening the fulfillment of the prophecy now made by scientists that the time will come when no man need die except by accident or design. —Medical Times.

